# Enhancing the Nutritional and Health-Related Properties of Taralli Through the Use of *Pleurotus eryngii*: Focus on Antioxidant and Anti-Inflammatory Properties

**DOI:** 10.3390/antiox14050550

**Published:** 2025-05-03

**Authors:** Giusy Rita Caponio, Graziana Difonzo, Marica Troilo, Ilaria Marcotuli, Agata Gadaleta, Grazia Tamma, Maria Letizia Gargano, Fortunato Cirlincione

**Affiliations:** 1 Department of Bioscience, Biotechnology and Environment, University of Bari Aldo Moro, Via Orabona 4, 70125 Bari, Italy; giusy.caponio@uniba.it (G.R.C.); grazia.tamma@uniba.it (G.T.); 2 Department of Soil, Plant and Food Sciences, University of Bari Aldo Moro, Via Amendola 165/A, 70126 Bari, Italy; marica.troilo@uniba.it (M.T.); ilaria.marcotuli@uniba.it (I.M.); agata.gadaleta@uniba.it (A.G.); marialetizia.gargano@uniba.it (M.L.G.); fortunato.cirlincione@uniba.it (F.C.)

**Keywords:** mushroom, dietary fibers, β-glucan, functional foods, antioxidant, anti-inflammatory

## Abstract

In this study, a portion of whole durum wheat semolina was replaced with *Pleurotus eryngii* powder (PeP) at concentrations of 5% and 10% (w/w) to produce two taralli variants, TPE5 and TPE10. The impact of PeP on the technological, chemical, physical, and sensory properties of taralli was evaluated. The functional characteristics of the enriched taralli were assessed employing HCT8 human colon carcinoma cells as the experimental model. The inclusion of PeP in taralli increased total dietary fiber, meeting the “high fiber” criteria under Regulation 1924/2006 while also enhancing the total phenol content. The higher fiber and polyphenol content in the enriched samples contributed to a significant reduction in glycemic index and starch hydrolysis. Treatment with PeP-enriched taralli resulted in a notable decrease in intracellular ROS levels in HCT8 cells, demonstrating strong antioxidant potential. Furthermore, TPE5 exerted beneficial effects by reducing inflammation—evidenced by a significant decrease in NFkB phosphorylation at serine 536—and by promoting apoptosis. These effects are likely mediated through the regulation of intracellular oxidative states. Overall, these findings indicate that PeP enrichment enhances the nutritional profile of taralli and provides potential health benefits, reinforcing its role as a valuable functional ingredient.

## 1. Introduction

In the last decade, the growing demand for foods with high nutritional value that promote health has encouraged the food industry to develop functional foods containing high concentrations of bioactive nutrients. According to the European Commission, functional foods are those that provide health benefits beyond basic nutrition, contributing to the prevention of disease and overall well-being [1]. At the same time, there has been growing interest in incorporating mushroom powders or extracts into commonly used foods due to their substantial health benefits, high nutritional value, and unique flavor and aroma [2]. Their functional properties have been demonstrated in several studies and specifically on baked products, such as breads, cakes, and pastries; the incorporation of mushroom influences several functional parameters, such as decreased glycemic index [3], raised polyphenolic content by enhancing antioxidant activity [4], and increased vitamin, mineral, fiber, and β-glucan content [5]. These compounds are associated with a wide range of therapeutic properties, including immune enhancement, antioxidant, antimicrobial and anti-inflammatory activities [6,7]. When incorporated into baked goods, mushrooms can provide both nutritional and functional benefits while enhancing the sensory qualities of the food, including flavor, texture, and aroma [8]. Traditional agrifood products are integral to the cultural identity of specific regions, acting as living representations of local agricultural practices, biodiversity, and food heritage. Taralli are small, crunchy, ring-shaped snacks that have become a symbol of Apulian cuisine. They are made from simple, locally sourced ingredients such as flour, oil, white wine, and salt, with various regional variations that usually include fennel [9]. Widely recognized as a crispy snack or bread substitute, taralli are highly versatile and have a long shelf-life [10]. *Agaricus bisporus* (J.E. Lange) Imbach (champignon), *Lentinula edodes* (Berk.) Pegler (shiitake), *Ganoderma lucidum* (Curtis) P. Karst. (reishi), and other medicinal mushrooms have been successfully used in the preparation of functional baked foods, i.e., *Pleurotus eryngii* (DC.) Quél. also included among Apulian traditional agri-food products under the local name of “cardoncello”. Nutritionally, *P. eryngii* is low in calories but rich in protein, fiber, and essential minerals such as potassium, phosphorus, and selenium. Scientific interest in *P. eryngii* has been growing due to its bioactive compounds, including antioxidants such as polyphenols and β-glucans, which have been linked to potential health benefits, particularly in anticancer activity and inflammation modulation [11]. Research has demonstrated that polysaccharides extracted from *P. eryngii* can inhibit tumor cell growth by activating the host immune system rather than directly targeting malignant cells [12,13]. In addition to β-glucans, *P. eryngii* contains phenolic compounds and ergothioneine, which endow the mushroom with natural antioxidant properties [14]. These antioxidants may help protect cells from oxidative stress, a key factor in tumor progression. Furthermore, the immunomodulatory effects of *P. eryngii*, particularly its high β-glucan content and potent genoprotective properties, have also been documented following in vitro fermentation by fecal inocula from healthy elderly volunteers [15]. With the aim of functionalizing an increasingly globally consumed baked product such as taralli, this study evaluated the impact of adding *P. eryngii* powder (PeP) to the formulation to improve its nutritional profile. The resulting products were analyzed for their main chemical, physical, sensory, and health-related aspects. In addition, taralli were subjected to glycaemic index evaluation and simulated gastro-intestinal digestion. Furthermore, this study investigated the functional effects of gastrointestinal digested taralli on HCT8 human colon cancer cells, focusing on cell viability and intracellular oxidative balance while also evaluating their anti-inflammatory properties through Western blot analysis.

## 2. Materials and Methods

### 2.1. Raw Materials

Wholemeal durum wheat semolina (La Molisana S.P.A., Campobasso, Italy), durum wheat semolina flour (Belbake, Arcole, Italy), sunflower oil (Desantis, Bitonto, Italy), white wine (Caviro, Faenza, Italy), bakery powder (Cameo s.p.a., Desenzano del Garda, Italy) and cooking salt were bought from the market. *P. eryngii* powder (PeP) was produced as reported by Cirlincione et al. [16] and provided by the Laboratory of Plant Defense of the Department of Agricultural, Food and Forest Sciences (SAAF), University of Palermo (Italy). In order to maximize the dietary fiber content of the taralli while maintaining a taste similar to that of the traditional product, the recipe was optimized using a mix of wholemeal semolina and semolina flour in a 1:1 ratio (w/w).

### 2.2. Dough Preparation

According to the experimental plan, three different trials were prepared (200 g). Control trial (CTR) was prepared using 121.1 g of semolina mix, 1.2 g of baking powder, 2.4 g of cooking salt, 30.5 g of sunflower oil, and 44.8 mL of white wine. For the preparation of the experimental trials, two different amounts of semolina mix were replaced with PeP, and 5% or 10% (w/w) were replaced to set up, respectively, TPE5 and TPE10 trials. The doughs were prepared using a planetary mixer mod. KHC29 (Kenwood limited, Havant, UK) mixing first dry ingredients followed by the liquid ingredients. After kneading, the dough was covered with plastic film, placed at 4 °C for 15 min, and then manually shaped. Taralli were cooked at 200 °C for 20 min in a static oven (Combo3, Zucchelli, Verona, Italy), and after cooling at room temperature, an aliquot was analyzed daily for the sensorial and textural profile analysis (TPA) while the other portion was vacuum-packed and frozen (−18 °C) until further analysis. Two productions were made within a month of each other.

### 2.3. Chemical and Nutritional Analyses of Taralli Samples

#### 2.3.1. Proximate Composition of Samples

The protein content (total nitrogen × 5.7) and the ash content were determined according to Association of Official Analytical Chemists methods (979.09 and 923.03, respectively). The total dietary fiber content was assessed using the enzymatic–gravimetric procedure as reported in method 985.29 [17], while lipid extraction was performed by Soxhlet apparatus using diethyl ether as solvent (method 945.38F, 2006). The carbohydrate fraction was estimated as a difference between protein, ash, moisture, and lipid contents from 100. All analyses were conducted in duplicate.

#### 2.3.2. Sample Extract Preparation

The extraction of polyphenols and antioxidant compounds was carried out following the method previously described [18], with specific modifications. In detail, 1 g of sample was extracted using 3 mL of an 80% methanol hydroalcoholic solution, subjected to an initial agitation phase for 10 min, followed by sonication treatment (CEIA international S.A., 115/230 Vac 1-50/60 Hz–400VA max, Viciomaggio, Italy) for 15 min. Subsequently, the extracts were shaken for an additional 30 min and centrifuged using a Thump SL 16R Thermo Scientific centrifuge for 10 min at 8000× *g* at 4 °C. The supernatants obtained were stored at −18 °C until further analysis. All extracts were prepared in duplicate.

#### 2.3.3. Total Phenol Content and Antioxidant Activity

The total phenol content (TPC) was quantified according to the Folin–Ciocalteu method as described by Difonzo et al. [19], with modifications. Specifically, 20 μL of filtered extract was mixed with 800 μL of Milli-Q water and 100 μL of Folin–Ciocalteu reagent. After 3 min of incubation, 800 µL of 7.5% Na_2_CO_3_ was added and then incubated in the dark for 60 min. Absorbance was measured with a Cary 60 spectrophotometer (Cernusco, Milan, Italy) at 720 nm. The results were expressed as mg of gallic acid equivalents (GAE)/g of sample.

The antioxidant activity was evaluated using the 2,2-diphenyl-1-picrylhydrazyl (DPPH), 2,2′-azino-bis(3-ethylbenzothiazoline-6-sulfonic acid) (ABTS), and Ferric reducing antioxidant power (FRAP) assays. For the DPPH test, 50 μL of sample was added to 950 μL of DPPH solution (0.08 mM in absolute ethanol; Carlo Erba, Val-de-Reuil, France), followed by 30 min of incubation in the dark. Absorbance was recorded at 517 nm. The ABTS assay involved the generation of ABTS ˙+ radicals through the reaction of 25 mL of ABTS solution (7 mM in H_2_O) with 800 μL of K_2_S_2_O_8_ (2.45 mM) in the dark for 16 h. The absorbance was measured at 734 nm after an 8 min incubation. The FRAP assay was performed according to the method of Razola-Díaz et al. [20] with modification. To 60 µL of sample, 180 µL of Milli-Q water was added, followed by 1800 µL of FRAP solution composed of 25 mL of 300 mM/L acetate buffer (pH 3.6), 2.5 mL of 20 mM/L FeCl3, and 2.5 mL of 10 mM/L TPTZ in 40 mM/L HCl. Absorbance was measured at 595 nm using a UV-visible spectrophotometer. The results of the ABTS, DPPH, and FRAP assays were expressed as µmol of Trolox equivalents (TE)/g of sample. All determinations were performed in triplicate.

#### 2.3.4. Determination of β-Glucan Content

For the determination of β-glucan content in semolinas and PeP, two different enzymatic procedures based on the spectrophotometric method were applied. Semolina’s samples were analyzed by using K-BGLU enzymatic assay kit (Neogen^®^’s Megazyme, Bray, Ireland) following the specific procedure for the quantification of 1,3:1,4-β-D-glucan mixed-linkage in cereal products [21]. The commercial enzymatic assay kit K-YBGL (Neogen^®^’s Megazyme, Bray, Ireland) was used for the quantification of 1,3:1,6-β-glucan in PeP, following the manufacturer’s instructions [22]. Both procedures involve the application of specific enzymes and conditions for the purification and hydrolysis of polysaccharides in the samples to release glucose. This, after reaction with GOPOD reagent, can be quantified via spectrophotometer by reading absorbance at 510 nm as reported in the producer guidelines. Due to the presence of starch, which interferes with the estimation of the content of fungal β-glucans in taralli, it was not possible to determine the content of these elements in the product, but the results obtained in the individual analyses have been reported as dry matter percentages [23].

### 2.4. In Vitro Starch Hydrolysis and Predicted Glycaemic Index

The in vitro starch hydrolysis of taralli was assessed following the methodology established by Difonzo et al. [24], aimed at simulation of the in vivo digestion of starch. In this process, 1 g of taralli samples was analyzed to determine their starch content. Hydrolyzed starch was measured by assessing the amount of glucose released, utilizing the D-glucose assay kit (GOPOD format) from Megazyme (Wicklow, Ireland). The starch content was calculated by taking the glucose quantification (mg) and multiplying it by a conversion factor of 0.9. To simulate digestion, the starch samples were subjected to dialysis through a membrane with a cut-off of 12,400 Da for 180 min. During this process, aliquots of the dialysate—rich in free glucose and partially hydrolyzed starch—were collected at 30 min intervals and treated with amyloglucosidase. Following this, the concentration of free glucose was determined using the enzyme-based kit described above, allowing for the conversion of the free glucose values into the amount of hydrolyzed (digested) starch content in taralli. To establish a baseline for comparison, control white wheat bread was employed as a reference point for estimating the hydrolysis index (HI), assigning it a value of 100. The predicted glycemic index (pGI) was then assessed using the equation: pGI = 0.549 × HI + 39.71, as outlined by Caponio et al. [25]. Each experimental sample was analyzed in triplicate to ensure accuracy and reliability of the results.

### 2.5. In Vitro Gastrointestinal Digestion of Taralli

A simulation was conducted to examine the impact of the gastrointestinal digestion tract on taralli, following the methods outlined by Caponio et al. [26]. The in vitro gastrointestinal digestion involved two phases: first, pepsin–HCl digestion was carried out for 3 h at 37 °C to simulate gastric digestion. This was followed by pancreatin digestion, which included pancreatin and bile salts for 3 h at the same temperature to simulate digestion in the small intestine. In brief, 10 mL of each extract were combined with 10 mL of an α-amylase solution at 56 mg/mL (Sigma-Aldrich Chemistry, St. Louis, MO, USA) and 10 mL of pepsin solution consisting of NaCl 125 mM/L, KCl 7 mM/L, NaHCO_3_ 45 mM/L, and pepsin 3 g/L (Sigma-Aldrich Chemistry, St. Louis, MO, USA). The pH of the solution was adjusted to 2 using hydrochloric acid, and the mixture was incubated at 37 °C for 180 min in a water bath while being shaken. After the incubation period, a portion of the gastric-digested extracts was set aside and stored at −20 °C for later analysis. The remaining extract was combined in equal volume with an intestinal solution, which was prepared by dissolving 0.1 g/100 mL of pancreatin (f Sigma-Aldrich Chemistry, St. Louis, MO, USA) and 0.15 g/100 mL of bile salts (Oxoid™, Hampshire, UK). The pH of the solution was adjusted to 8 using NaOH and incubated at 37 °C for 180 min in a water bath under shaking. Samples were collected at the conclusion of the gastric phase (GP) and at the conclusion of the duodenal phase (DP). For the GP samples, the pH was raised to 8 with 35% NaOH to stop the hydrolytic action of pepsin, and then it was adjusted back to 2 with 37% HCl. The samples at DP were acidified to pH 2 using 37% HCl to stop pancreatic hydrolysis, and then the pH was elevated to 8 with 35% NaOH. The digested samples were centrifuged at 18,500× *g* for 20 min, and the upper oil phase was discarded. To eliminate any turbidity, the supernatant of the aqueous micellar phases was filtered through 0.2 μm cellulose acetate membranes and stored at −80 °C until further analysis. Each sample digestion was performed in triplicate, and the triplicate was then combined for analysis.

### 2.6. Colour and Texture Analyses

Colorimetric analysis was performed through a CM-600d colorimeter (Konica Minolta, Tokyo, Japan) provided with SpectraMagic NX software (Konica Minolta, Tokyo, Japan). Lightness (L*, ±black-white), redness (a*, ±red-green), and yellowness (b*, ±yellow-blue) were measured as color parameters. Twelve replicates of each analysis were carried out. Additionally, the brown index (BI) [27], which represents the browning tendency on a scale from 0 to 100, and total color difference (∆E) were calculated as reported:

$$∆E=\sqrt{\left[{\left(L^{*}-L_{0}^{*}\right)}^{2}+{\left(a^{*}-a_{0}^{*}\right)}^{2}+{\left(b^{*}-b_{0}^{*}\right)}^{2}\right]}$$

$$BI=100-L^{*}$$

where *L*_0_*, *a*_0_*, *b*_0_* represent the color coordinates of control trial (CTR), while L*, b*, and a* are the color coordinates of the fortified ones. For result interpretation, the following ∆E scale was used: 0–0.5 = no relevant difference; 0.5–1.5 = a slight difference; 1.5–3.0 = difference noticeable by an experienced observer; 3.0–6.0 = an appreciable difference; 6.0–12.0 = a large difference; and >12.0 = an evident difference [28].

Textural properties were analyzed using a three-point bending test as described in Vurro et al. [29] using a Texture Analyzer Z1.0 TN equipped with a 1 kN load cell (Zwick Roell, Ulm, Germany). The support bars were 3 cm apart, while the probe, moving at a speed of 5 mm/min, descended until the taralli fractured. The maximum force required to break the samples and the probe displacement at the breaking point, representing hardness (N/mm^2^) and fracturability (mm), respectively, were recorded. The analysis was performed in nine replicates.

### 2.7. Sensory Evaluation

Sensory evaluation was conducted following the ethical guidelines of the Laboratory of Food Science and Technology of the Department of Plant, Soil, and Food Science at the University of Bari Aldo Moro, Italy. The evaluation was carried out following the UNI EN ISO guidelines 13299 [30] and involved 11 semi-trained judges aged between 24 and 58 years. Panelists were informed about the study’s objectives and provided written consent to participate in the sensory analysis, in line with the ethical guidelines of the laboratory. Pre-test sessions were held to establish a list of descriptors and to assess the judges’ ability to discriminate, as well as their consistency and reliability. The samples were randomized and presented to the panelists in white dishes marked with alphanumeric codes. The evaluated descriptors (11) focused on visual elements (color intensity and homogeneity), tactile analysis (friability and hardness), gustatory characteristics (salty, bitterness, astringency, and taste intensity), olfactory sensation (odor intensity), and off taste and flavor intensity.

### 2.8. In Vitro Assays of Digested Samples on Cell Cultures

#### 2.8.1. Cell Culture and Treatments

The human ileocecal adenocarcinoma cell line, HCT8, was cultured according to previously established protocols [31]. Briefly, the cells were multiplied in Advanced RPMI-1640 supplemented with 10% fetal bovine serum (FBS), 100 i.u. per mL penicillin and 100 μg/mL streptomycin at 37 °C in 5% CO_2_. Cells were subjected either to basal condition (untreated) or treated with digested taralli extract at increasing concentration series (1:400; 1:200; 1:150; 1:100; 1:50; 1:25) for 24 h.

#### 2.8.2. Antibodies

NFkB (1:200), NFkB-pS536 (1:200), and BID (1:200) were acquired by Santa Cruz Biotechnology (Santa Cruz, CA, USA).

#### 2.8.3. Calcein-AM Cell Viability Assay

Calcein-AM is a non-fluorescent compound known for its ability to easily penetrate cell membranes. In viable cells, intracellular esterase converts calcein-AM into a green fluorescent compound that cannot pass through the plasma membrane, causing it to accumulate in live cells with intact membranes. HCT8 cells were treated as previously described. After treatment, the cells were incubated with calcein-AM (1 μM) at 37 °C for 45 min, after which the fluorescence signal was assessed and examined. As an internal positive control, cells were incubated with ethanol (90%) for 1 min.

#### 2.8.4. ROS Detection

ROS were detected, as already shown [32]. After treatments, cells were incubated with dihydro rhodamine-123 (10 μM) at 37 °C for 30 min and then recovered in a complete medium for 30 min. Cells were lysed in RIPA buffer containing 150 mM NaCl, 10 mM Tris-HCl pH 7.2, 0.1% SDS, 1.0% Triton X-100, 1% sodium deoxycholate, and 5 mM EDTA. Samples were centrifuged at 12,000× *g* for 10 min at 4 °C, and the supernatants were used for ROS detection. As a positive control, cells were treated with tert-butyl hydroperoxide (tBHP, 2 mM for 30 min). The fluorescence emission signal was recorded using a fluorimeter FLUOstar Omega (BMG LABTECH, Offenburg, Germany) at excitation and emission wavelengths of 508 and 529 nm, respectively.

#### 2.8.5. Cell Lysates and Western Blotting

Human colon HCT8 cells are originated from a 67-year-old male patient with ileocecal adenocarcinoma (ATTC). HCT8 cells were plated onto 60 mm diameter Petri dishes, treated with digested extracts and lysed in RIPA buffer containing 150 mM NaCl, 10 mM Tris-HCl pH 7.2, 0.1% SDS, 1% Triton X-100, 1% deoxycholate and 5 mM EDTA, 1 mM PMSF, 2 mg mL^−1^ leupeptin and 2 mg mL^−1^ pepstatin, 10 mM NaF, and 1 mM sodium orthovanadate. Cellular debris was eliminated by centrifugation at 12,000× *g* for 10 min at 4 °C. The resulting supernatants were gathered and used for the western blotting analyses. Proteins were separated using 10% or 12% stain-free polyacrylamide gels (Bio-Rad Laboratories, Inc., Hercules, CA, USA). Following, the protein bands were electrophoretically transferred onto PVDF Immobilon-P membrane (Bio-Rad Laboratories, Inc., Hercules, CA, USA) and incubated with EveryBlot blocking solution (Bio-Rad Laboratories, Inc., Hercules, CA, USA). Blots were then incubated overnight with primary antibodies. To detect the immunoreactive bands, secondary anti-mouse horseradish peroxidase-coupled antibodies (Bio-Rad Laboratories, Inc., Hercules, CA, USA) were utilized. Membranes were subsequently treated with Clarity^TM^ Western ECL Substrate (Bio-Rad Laboratories, Hercules, CA, USA), and the signals were displayed with the ChemiDoc System gels (Bio-Rad Laboratories, Inc., Hercules, CA, USA). The resulting bands were normalized to total protein using stain-free technology gels (Bio-Rad Laboratories, Inc., Hercules, CA, USA). Densitometry analysis was conducted with Image Lab gels (Bio-Rad Laboratories, Inc., Hercules, CA, USA).

### 2.9. Statistical Analysis

The results were reported as the mean ± standard deviation (SD). Significant differences (*p* ≤ 0.05) were assessed using unidirectional analysis of variance (ANOVA), followed by Tukey test for multiple comparisons. The statistical analysis was performed using Minitab 17 (Minitab Inc., State College, PA, USA) statistical software.

## 3. Results

### 3.1. Chemical and Nutritional Characterization of Taralli Samples

#### 3.1.1. Nutritional Composition

Table 1 shows the nutritional profile of the different taralli formulations. The results showed a slight decrease in moisture content, which dropped from 3.58% in CTR to 3.18% in TPE10 formulation, in line with findings reported by Owheruo et al. [33] and Slawińska et al. [34]. However, the same authors observed variations in protein content, with a proportional increase with the increased percentage of mushroom flour added, attributable to its higher concentration. In the samples reported in the table, the addition of 5% and 10% of PeP did not significantly affect protein content (8.94% and 8.78%, respectively) compared to the control (9.04%). The increase in mushroom powder content from 0% to 10% contributed to a significant increase (*p* < 0.05) in lipid concentration (20.2% vs. 21.07% for CTR and TPE10, respectively) and total dietary fiber (4.67% vs. 7.91%), accompanied by a decrease in total carbohydrates. This trend is consistent with previous studies on cookies and bread enriched with mushroom powder, as reported by Biao et al. [35], Farooq et al. [36], Losoya-Sifuentes et al. [37], and Slawińska et al. [34]. The incorporation of mushroom flour in the taralli production process proved to be an effective strategy for improving their nutritional profile. Although increasing PeP in the dough led to a proportional rise in lipid content, the powder, as highlighted by Losoya-Sifuentes et al. [37], is rich in monounsaturated and polyunsaturated fatty acids, whose consumption is associated with potential health benefits. Moreover, the substitution of 10% durum wheat semolina determined a significant increase in fiber content, which doubled compared to the control trial, accompanied by a slight reduction in carbohydrate content in the fortified ones. This result emphasized the positive contribution of mushroom flour to the formulation due to its richness in complex polysaccharides, including β-glucans, chitin, and hemicellulose [38]. Given that the PeP-enriched samples contained more than 6.52% and 7.91% of total dietary fiber, in accordance with the guidelines of the EC Regulation No. 1924/2006 [39], the fortified samples can be labeled with the nutritional claim “high fiber content”, as they provide more than 6 g of total dietary fiber per 100g of product. This increase is primarily due to the presence of polysaccharides in cereals and mushrooms, particularly β-glucans, which contribute to the dietary fiber content [40]. A similar trend was observed in β-glucans content, which significantly increased (*p* < 0.001) from 0.15 ± 0.01 g/100 g in CTR to 3.05 ± 0.21 g/100 g TPE10 detecting an intermediate value of 1.61 ± 0.11 g/100 g in TPE5. These results align with previous studies that demonstrate the enrichment potential of *P. eryngii* in functional foods [5,41]. The increase in β-glucans content is particularly relevant given their well-documented health benefits, including cholesterol reduction, improved glycemic response, and immune modulation. It is important to highlight the structural differences between β-glucans derived from cereals and those from mushrooms. While cereal β-glucans primarily feature (1–3),(1–4) glycosidic linkages, mushroom β-glucans contain (1–3),(1–6) linkages, which enhance their biological activity [42]. This distinction suggests that the incorporation of mushroom-derived β-glucans into wheat-based products not only enhances dietary fiber content but may also provide additional functional health benefits, such as improved immune response and antioxidant properties. Recent studies have highlighted the potential of PeP as a valuable ingredient in the development of functional foods aimed at improving human health. For instance, incorporating PeP into wheat flour products has been shown to enhance their nutritional value, suggesting its utility in functional food formulations [43].

Studies have indicated that β-glucans from edible mushrooms may influence gut microbiota composition, which is crucial for maintaining immune and metabolic homeostasis [44]. Additionally, the structural characteristics of mushroom β-glucans, such as those from *P. eryngii*, may contribute to their bioactivity and potential health benefits. Therefore, comprehensive studies focusing on their bioavailability, interactions with other dietary components, and effects on gut microbiota are essential to fully harness their potential in functional food applications [43,44], making them a more balanced source of macronutrients. The formulations with mushroom powder also promoted an increase in ash content, with values of 2.58 ± 0.05 g/100 g and 3.09 ± 0.05 g/100 g for the control and TPE10 samples, respectively, attributed to the higher presence of minerals such as potassium, iron, phosphorus, copper, and zinc [33,37,45]. Finally, the energy value showed slight but statistically significant differences among formulations. In particular, the control samples showed values of 467.02 Kcal, compared to 464.45 Kcal in the 10% mushroom powder formulation, likely due to the low energy content of the mushroom powder. The growing interest in functional foods has led to the exploration of alternative ingredients that enhance both the nutritional profile and health benefits of staple products. These findings emphasize the potential of PeP as a valuable ingredient in the development of functional foods aimed at improving human health. However, further research is needed to explore the bioavailability and physiological effects of mushroom-derived β-glucans in fortified products. Understanding their interactions with other food components and their impact on gut microbiota could provide further insights into their role in promoting overall well-being.

#### 3.1.2. Phenolic Compounds and Antioxidant Activity

**Figure 1:**
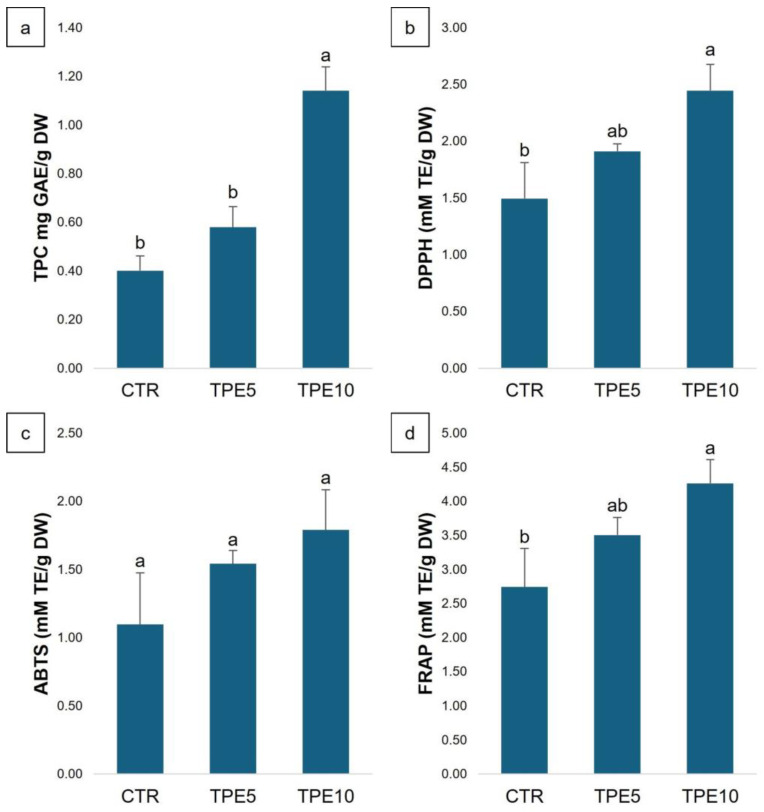
The total phenol content (TPC) (**a**), DPPH (**b**), ABTS (**c**), and FRAP (**d**) assays of taralli. Data are shown as mean of triplicates ± SD and analyzed by one-way ANOVA followed by Tukey’s multiple comparison test. Different small letters above the column showed significant difference between trials (*p* < 0.05). Abbreviations: CTR, control trial; TPE5, taralli with 5% of PeP (w/w); TPE10 taralli with 10% of PeP (w/w).

Medicinal mushrooms are rich in bioactive molecules that may have beneficial health properties, and among these, the most studied are polyphenols, which possess antioxidant activity [46]. Several studies have shown that adding mushroom powder to bakery products consistently increases their phenolic content, with the effect varying by mushroom species and the concentration used. The results of TPC and antioxidant activities are reported in Figure 1. As expected, the TPC value (Figure 1a) increases with increasing amount of PeP added, from 0.40 ± 0.06 mg GAE/g DW in the CTR trial to 1.14 ± 0.10 mg GAE/g DW in TPE10. Similarly, the three tests performed to assess antioxidant activity (DPPH, FRAP, and ABTS) also showed similar increases in TPC. The DPPH radical scavenging capacity (Figure 1b) and FRAP (Figure 1d) were significantly higher (*p* < 0.05) in TPE10 (2.45 ± 0.23 and 4.27 ± 0.35 mM TE/g DW, respectively) than in the CTR trial (1.5 ± 0.32 and 2.74 ± 0.57 mM TE/g DW, respectively). These results are consistent with those reported in other studies. As reported by Sławińska et al. [34], replacing wheat flour with freeze-dried mushroom powder in shortbread-type cookies leads to an increased TPC value and enhanced antioxidant ability. This is also emphasized in a recent study by Kobus et al. [47], which evaluates the biological activities of gluten-free bread enriched with dried chaga mushroom [*Inonotus obliquus* (Fr.) Pilát] powder. Regarding the ABTS assay (Figure 1c), although increases in antioxidant activity were observed, the differences found were not significant. This occurs because the DPPH assay is related to phenolic compounds, while the ABTS assay correlates more with flavonoid content [48].

### 3.2. In Vitro Starch Hydrolysis and Predicted Glycemic Index

**Figure 2:**
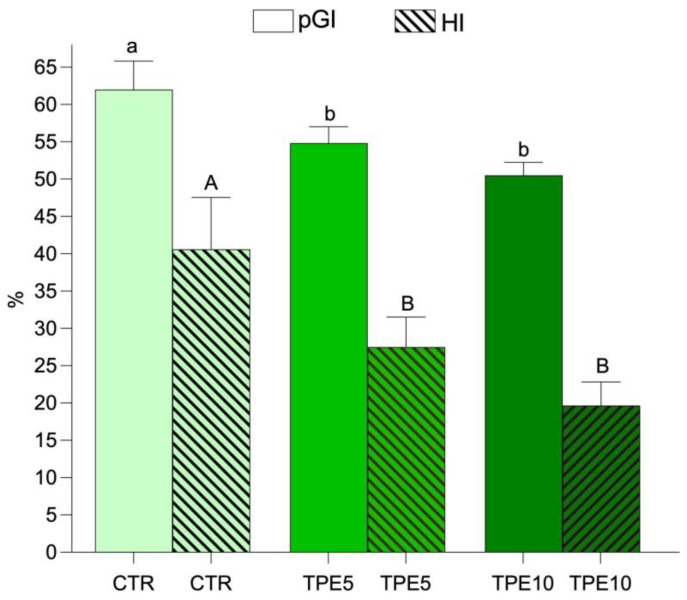
Results of predicted glycemic index (pGI) and hydrolysis index (HI) of biscuits. Data are shown as mean of triplicates ± SD and analyzed by one-way ANOVA followed by Tukey’s multiple comparison test. Different lowercase and uppercase letters indicate a significant difference (*p* ≤ 0.05) among samples for pGI and HI, respectively. Abbreviations: CTR, control trial; TPE5, taralli with 5% of PeP (w/w); TPE10 taralli with 10% of PeP (w/w).

Mushroom flour is an innovative ingredient in the food industry, commonly used to enhance various foods, particularly baked goods and protein-rich products. Interest in this flour is growing due to its natural content of protein, fiber, and micronutrients such as B vitamins, zinc, and selenium. Additionally, mushroom flour is notable for its β-glucan content, a type of fiber that contributes to maintaining a low glycemic index. In this study, we explored the potential of taralli with added mushroom flour by evaluating their glycemic index in vitro. Specifically, the taralli were tested to determine their predicted glycemic index (pGI) (represented by the smooth bar in Figure 2) and hydrolysis index (HI) (represented by the textured bar in Figure 2) across different formulations. As shown in Figure 2, the control sample had a HI value of approximately 40.59% ± 6.96 and a pGI, calculated from the HI, of about 62% ± 3.82. Taralli with 5% (TPE5) and 10% (TPE10) mushroom flour had pGI values ranging from 54.81% ± 2.20 to 50.51% ± 1.73, respectively. Although the differences were small, a significant downward trend in proportional pGI values was observed with the addition of mushroom flour. Both the TPE5 and TPE10 samples exhibited lower pGI values compared to the CTR control. According to the International Organization for Standardization (ISO), foods are classified based on their glycemic index as high GI (GI ≥ 70), medium GI (GI 56–69), or low GI (GI ≤ 55) [49]. Notably, taralli made with 5% and 10% mushroom flour (TPE5 and TPE10) achieved a pGI level that qualifies them as low GI.

This effect is nutritionally significant and aligns with the growing interest in functional ingredients that can help stabilize blood sugar, particularly in baked goods, which are typically high in glycemic index. The literature supports the role of β-glucans, found in mushroom flour, in lowering the glycemic index of foods. Numerous studies have shown that β-glucans slow carbohydrate absorption during digestion, positively affecting postprandial blood sugar levels. Flours containing β-glucans, such as those from oats or mushrooms, are associated with reduced glycemic index in baked goods due to the fiber’s viscosity and its ability to moderate the insulin response [40,50]. Additionally, baked goods like taralli are typically high in refined carbohydrates, which lead to a rapid spike in blood sugar levels. Research indicates that incorporating alternative flours rich in fiber and protein can lower the glycemic load of these products. For instance, studies on legume flour and other fiber-rich ingredients demonstrate a similar effect [51]. The results align with previous research findings [52] demonstrating a pGI reduction of up to 50% in samples containing 10% mushroom flour. This suggests that mushroom flour may play a similar role in lowering the glycemic index, supporting its use as an alternative flour to reduce the glycemic impact of baked goods. Compared to other alternative flours aimed at reducing pGI, such as almond or coconut flour, mushroom flour stands out due to its low fat content and high levels of soluble fiber. The literature indicates that flours high in fiber, protein, and bioactive compounds (like β-glucans) have a cumulative effect in lowering pGI [53]. According to ISO classification [49], a diet primarily comprising low- and medium-glycemic index foods can improve glycemic control and reduce the risk of metabolic diseases [54]. The finding that mushroom flour taralli can be classified within the low-glycemic index range underscores the potential of mushroom flour as a highly effective ingredient for developing healthier baked goods. This result is particularly significant for the functional food industry, as it provides consumers with snack options that have a lower glycemic impact, supporting blood sugar regulation and weight management. In conclusion, the study’s findings confirm the beneficial effect of mushroom flour in reducing the glycemic index of taralli, aligning with the existing literature on the effectiveness of β-glucans and dietary fiber in modulating glycemic responses. Mushroom flour taralli could represent a promising alternative for health-conscious consumers, especially those managing blood sugar levels or adhering to a low-GI diet.

### 3.3. Texture and Color Profile of Taralli Samples

The texture analysis, performed using the three-point bending test, showed that the addition of 5% *P. eryngii* flour resulted in the lowest hardness (21.26 ± 1.09 N/mm^2^) and fracturability (0.04 ± 0.03 mm) values compared to the control (CTR) and to the 10% PeP-enriched sample (TPE10), which exhibited similar values (Table 2). These findings suggest that both the fortification level and the intrinsic properties of mushroom flour critically influence the final structure of products due to its high total dietary fiber content, which, as reported by Sławińska et al. [34], tends to increase food hardness. Notably, the specific nature of fiber, particularly β-glucans, appears to be a key factor. At low concentrations (5%), these compounds may act as structural modifiers by enhancing water and protein distribution in the dough, thus increasing plasticity and reducing mechanical resistance, which improves fracturability. However, at higher concentrations (10%), excessive fiber content may interfere with gluten network formation, disrupting its continuity and increasing hardness, as observed by Rathore et al. [55] in biscuits fortified with Calocybe indica powder, although statistically significant changes were noted only when the substitution exceeded 5%. Similarly, experiments conducted by Gaglio et al. [56] on bread fortified with *P. eryngii* demonstrated an increase in firmness with a higher concentration of added mushroom powder. In contrast, studies by Chen et al. [57] and Shams et al. [58] reported decreasing hardness values with increasing amounts of mushroom flour in biscuits fortified with *Cordyceps militaris* and *A. bisporus*, suggesting that the impact of mushroom flour on texture may be influenced by the specific mushroom species used. In addition, the competition for water between β-glucans, starch, and gluten may compromise optimal hydration, leading to a less elastic and more rigid dough.

Color is a crucial factor influencing consumer choice, acceptance, and preference in food products. Table 2 presents the color profile for lightness (L*), redness (a*), and yellowness (b*) indices of taralli. Lightness significantly decreased (*p* < 0.05) as the mushroom powder content increased, from 52.03 in CTR to 40.68 in TPE10; in fact, control samples had a typical golden shade, characteristic of traditional baked goods, compared to the fortified ones that appeared darker and brownish. This difference can be attributed both to the increase in total dietary fiber, as suggested by Difonzo et al. [19], and to the presence of natural pigments in mushroom flour [57,59]. In bakery products, color variations are often linked to chemical reactions induced by the baking process, such as the Maillard reaction and caramelization, both of which contribute to increased browning [37,57,58]. Specifically, the brown coloration observed in TPE5 and TPE10 samples can be attributed to the formation of melanoidins—high-molecular-weight brown pigments—during the final stage of the Maillard reaction, which involves the condensation of reducing sugars and amino acids at high temperatures [34]. This phenomenon is further supported by BI values, which showed a progressive increase in the browning index as PeP concentrations increased compared to CTR. Likewise, b* progressively decreased in experimental samples, indicating a bluish hue as the amount of PeP increased. Similar results were reported by Miranda et al. [60] and Stoffel et al. [61] in cookies fortified with *P. albidus* and snacks containing cactus pear peel powder, respectively. In contrast, redness exhibited an opposite trend, with higher values in fortified samples (14.52 and 14.61 for TPE5 and TPE10, respectively) compared to CTR (13.78). Finally, the total color difference (ΔE) between the experimental samples and control exceeded five, indicating significant chromatic differences [62]. This result reflects a direct correlation between the amount of added mushroom flour and the overall color variation of the product, showing that all experimental trials were clearly differentiated from control [57].

### 3.4. Sensory Analysis

**Figure 3:**
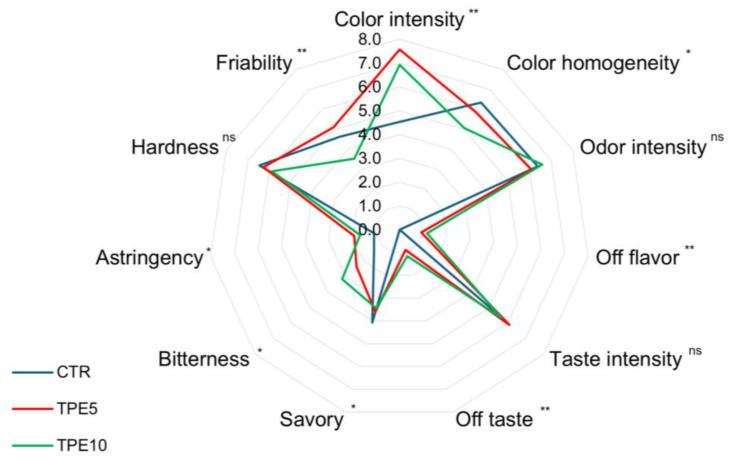
Spider plots of descriptive sensorial analysis of Taralli. Data are shown as mean of the values assigned and analyzed by one-way ANOVA followed by Tukey’s multiple comparison test. *p* value: *, *p* < 0.05; **, *p* < 0.01; ns, not significant. Abbreviations: CTR, control trial; TPE5, taralli with 5% of PeP (w/w); TPE10 taralli with 10% of PeP (w/w).

Sensory evaluation is crucial for the development of innovative foods as they may have a lower acceptability than traditional ones. The scores obtained by the different trials are reported in the spider plot in Figure 3. Regarding visual descriptors, TPE5 and TPE10 achieved significant (*p* < 0.001) higher values in terms of color intensity (7.60 and 6.92, respectively) than the control trial (4.53). This result agrees with previous work focused on bakery products [5] and may be due to the pigments contained in the mushrooms’ cell walls [63] and to chemical reactions occurring during cooking between sugars, proteins, and phenolic compounds of mushrooms, which could alter the final color of the product [64]. The olfactory analysis showed no significant differences in the overall intensity of the aromas between the samples, but in TPE5 and TPE10, slight notes of off odors were found, which were statistically different from the CTR trial (*p* < 0.001). In the same way, the retro-olfactory analysis showed no significant differences in overall flavor intensity, but in the mushroom-containing sample, significant perception of an off flavor (*p* < 0.001), bitterness, and astringent sensations were found (*p* < 0.05). These characteristics are common in mushroom-added baked products [56,65] since, as mentioned above, they possess several compounds able to confer unique flavors, often atypical for baked goods. Additionally, the phenolic compounds present in PeP are known to contribute to bitterness and astringency sensations [66]. In conclusion, the overall assessment of the product showed that, although the CTR sample was the most appreciated, the overall acceptance of TPE5 and TPE10 showed no significant differences.

### 3.5. In Vitro Characterization of Samples on Cell Cultures

#### 3.5.1. Cell Viability and ROS Detection

The effect of scalar dilutions of digested sample formulations on cell viability is shown in Table 3. Specifically, HCT8 cells were either left untreated (CTR−) or treated with different dilution series (dilutions of 1:400, 1:200, 1:150, 1:100, 1:50, and 1:25) of the digested samples for 24 h. As a positive control (CTR+), cells were treated with 90% ethanol for 1 min. At the lowest concentration tested (1:400 dilution), only the TPE5 sample showed a significant reduction in cell viability compared to untreated cells, and this trend persisted as the concentration increased (1:200, 1:150, and 1:100 dilutions). At a dilution of 1:50, both mushroom flour-supplemented samples demonstrated a reduction in cell viability. At the highest concentration tested (1:25 dilution), all taralli formulations significantly reduced cell survival. Based on these findings, a dilution of 1:100 was selected for subsequent experiments.

**Figure 4:**
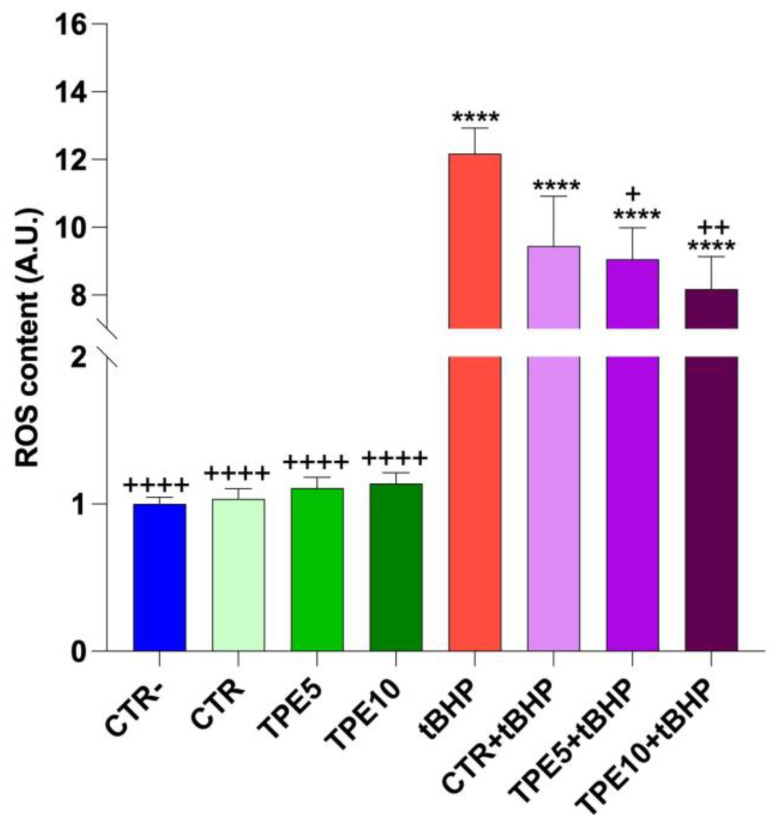
ROS content measured using dihydrorhodamine-123 fluorescence in HCT8 cells treated for 24 h at dilution 1:100 of digested samples. Data are shown as mean ± SEMs and analyzed by one-way ANOVA followed by Tukey’s multiple comparison test. **** means a significant difference (*p* ≤ 0.0001) of samples vs. CTR−; +, ++, ++++ mean a significant difference (*p* ≤ 0.05, *p* ≤ 0.005, *p* ≤ 0.0001, respectively) of samples vs. tBHP. Abbreviations: CTR, control trial; TPE5, taralli with 5% of PeP (w/w); TPE10 taralli with 10% of PeP (w/w).

To assess the antioxidant activity of the digested taralli extract, HCT8 colorectal carcinoma cells were treated as previously described, and intracellular ROS content was measured. As shown in Figure 4, the digested taralli extract did not significantly increase ROS levels in HCT8 colorectal carcinoma cells, suggesting that the extract’s compounds do not have a pro-oxidant effect under basal conditions. However, compared to tert-butyl hydroperoxide (tBHP), a synthetic pro-oxidant compound, incubation with both TPE5 and TPE10 extracts significantly reduced intracellular ROS levels induced by tBHP, indicating a clear antioxidant effect of the extracts. This trend was in line with the results of antioxidant activity shown in Figure 1.

The research literature supports the idea that fibers in mushrooms, particularly β-glucans, possess notable antioxidant properties. Several studies have demonstrated that mushroom extracts, such as those from Pleurotus, contain natural antioxidants that effectively reduce intracellular ROS levels, thereby protecting cells from oxidative stress [67,68]. The reduction in ROS observed in the presence of tBHP with the TPE5 and TPE10 samples is consistent with previous research indicating that mushroom extracts can mitigate oxidative stress triggered by pro-oxidant agents. As a known inducer of reactive oxygen species, tBHP generally leads to cellular damage; however, the application of antioxidant compounds has been shown to counteract this effect. Antioxidants found in mushrooms, including polyphenols and other bioactive molecules, can neutralize free radicals, thus lowering lipid peroxidation and reducing oxidative cellular damage, contributing to an overall protective effect [69,70]. In conclusion, these results suggest that mushroom flour, particularly at 5% and 10% concentration, provides antioxidant benefits capable of mitigating ROS increases caused by pro-oxidants such as tBHP. This finding aligns with the existing literature on the antioxidant activity of mushrooms and β-glucans and indicates that taralli enriched with mushroom flour could be a valuable functional snack, potentially delivering health benefits by decreasing oxidative stress and reducing the risk of cellular damage.

#### 3.5.2. Western Blotting Analysis

**Figure 5:**
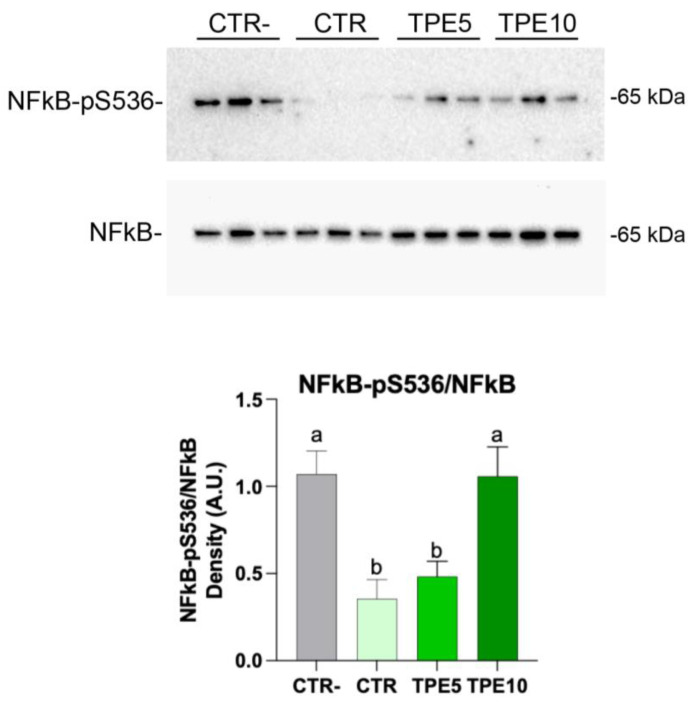
Western blotting analysis of proteins in HCT8 cells exposed with digested extract for 24 h for NFkB-pS536, NFkB, and relative ratio. Data are shown as mean ± SEMs and analyzed by one-way ANOVA followed by followed by Tukey’s multiple comparison test. Different letters indicate a significant difference (*p* ≤ 0.05) among samples. Abbreviations: CTR, control trial; TPE5, taralli with 5% of PeP (w/w); TPE10 taralli with 10% of PeP (w/w).

Oxidative stress, driven by the production of ROS, plays a critical role in inflammatory processes. We hypothesized that the digested taralli could modulate ROS levels, thereby offering protection against oxidative stress and inflammation. NFkB signaling is a key pathogenic factor in numerous inflammatory diseases [71,72] and is known to mediate the production of inflammatory cytokines such as TNF-α, IL-1, and IL-6 [73]. To explore whether the anti-inflammatory effects of the digested taralli occur via NFkB signaling, we analyzed the expression and the phosphorylation level of NFkB (p65). Increased phosphorylation of NFkB at serine 536 activates the NFkB-induced inflammation signals [74]. Here, Figure 5 (Figures S1 and S2) showed that compared to untreated cells, cells treated with extract obtained from control taralli (CTR) and the extract from the formulation containing 5% of mushroom flour (TPE5) displayed a significant decrease in NFkB phosphorylation at serine 536 consistent with a significant downregulation of the NFkB inflammatory functions. By contrast, the TPE10 sample did not evoke an anti-inflammatory response, exhibiting values comparable to untreated cells (CTR-). These findings, indeed, underline that the TPE5 formulation reduced oxidative stress and inflammation, as evidenced by a decreasing trend in ROS levels.

**Figure 6:**
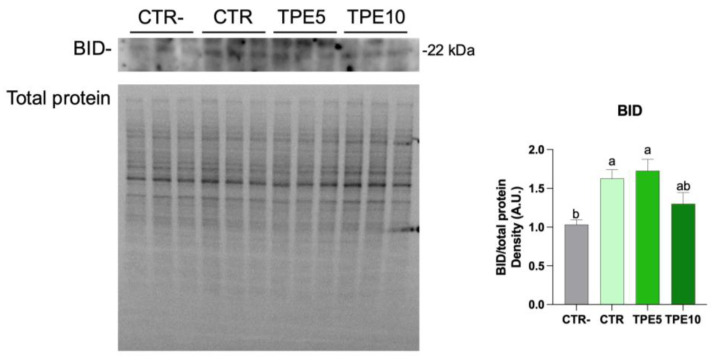
Western blotting analysis of proteins in HCT8 cells exposed with digested extract for 24 h for BID and relative ratio. Data are shown as mean ± SEMs and analyzed by one-way ANOVA followed by followed by Tukey’s multiple comparison test. Different letters indicate a significant difference (*p* ≤ 0.05) among samples. Abbreviations: CTR, control trial; TPE5, taralli with 5% of Pep (w/w); TPE10 taralli with 10% of PeP (w/w).

It is well established that NFkB is involved in several functions and pathways [74], including cellular transformation and tumorigenesis, where it suppresses apoptotic pathways [75]. Moreover, NFkB inhibits apoptosis by upregulating the expression of Bcl-2, an anti-apoptotic protein [76]. Conversely, inhibition of NFkB phosphorylation is correlated with increased apoptosis. Therefore, to evaluate whether inhibition of NFkB phosphorylation affects cell apoptosis, we examined the expression of BID, a pro-apoptotic member of the Bcl-2 family, following treatment with taralli digest extracts. Western blot analysis likely indicated an enhanced apoptotic response in HCT8 cells, as evidenced by increased BID levels upon exposure to the digested taralli samples (Figure 6 and Figure S3). This response was particularly prominent in the CTR and TPE5 samples. Conversely, the TPE10 sample exhibited a reduced apoptotic response, likely due to heightened NFkB expression, consistent with prior studies [77]. On the other hand, it cannot be excluded that in cells exposed to TPE10, proliferative signals are induced, thereby preserving cell survival. Conversely, compared to CTR samples, we may speculate that TPE5 treatment exhibited beneficial action, exerting anti-inflammatory effects and promoting cell apoptosis of adenocarcinoma HCT8 cells, possibly by modulating the intracellular oxidative states.

## 4. Conclusions

The incorporation of PeP into taralli significantly improved the nutritional and functional profile of the product. The increase in dietary fiber qualifies it for the “high fiber” designation according to Regulation 1924/2006 [39], while the high total phenolic content and antioxidant activity provide additional health benefits. From a functional perspective, the reduction in pGI and HI highlights the potential of PeP in modulating the glycemic response, making it a promising ingredient for low-glycemic index foods. To further investigate the functional mechanism and the molecular players modulated by PeP, adenocarcinoma human colon HCT8 cells were used as an experimental model. Importantly, we found that PeP can modulate the expression and functionality of NFkB and BID, which are paramount markers of key cellular processes such as inflammation and apoptosis. Here, we propose that PeP prevents oxidative stress, reduces NFkB activity, and promotes cell apoptosis, which is often downregulated in tumor cells. Therefore, the integration of *P. eryngii* into durum wheat taralli offers a dual benefit: enhancing the health-promoting properties of the product while maintaining its appeal as a tasty snack. With growing consumer demand for functional foods, PeP-enriched baked goods could represent a valuable option for the nutrition-conscious market, contributing to improved health outcomes and overall consumer well-being.

## Figures and Tables

**Table 1:** Nutritional composition and energy value (g/100 g).

	CTR	TPE5	TPE10
Energy (kcal)	467.02 ± 0.15 ^a^	466.63 ± 0.90 ^a^	464.45 ± 0.55 ^b^
Moisture	3.58 ± 0.20 ^a^	3.27 ± 0.03 ^ab^	3.18 ± 0.15 ^b^
Protein	9.04 ± 0.05 ^a^	8.94 ± 0.09 ^a^	8.78 ± 0.37 ^a^
Lipid	20.2 ± 0.09 ^b^	20.99 ± 0.09 ^a^	21.07 ± 0.01 ^a^
Total carbohydrate	64.6 ± 0.02 ^a^	63.75 ± 0.04 ^b^	63.88 ± 0.24 ^b^
Dietary fiber	4.67 ± 0.01 ^c^	6.52 ± 0.02 ^b^	7.91 ± 0.04 ^a^
β-glucans	0.15 ± 0.01 ^c^	1.61 ± 0.11 ^b^	3.03 ± 0.21 ^a^
Ash	2.58 ± 0.05 ^b^	3.05 ± 0.14 ^a^	3.09 ± 0.02 ^a^

Data are reported as SD mean and analyzed by one-way ANOVA followed by Tukey test for multiple comparisons. Different letters in row differ significantly with *p* < 0.05. Abbreviations: CTR, control trial; TPE5, taralli with 5% of PeP (w/w); TPE10 taralli with 10% of PeP (w/w).

**Table 2:** Texture analysis and color profile of taralli.

	CTR	TPE5	TPE10
Hardness (N/mm^2^)	30.22 ± 0.67 ^a^	21.26 ± 1.09 ^b^	31.59 ± 0.99 ^a^
Fracturability (mm)	0.13 ± 0.02 ^a^	0.04 ± 0.03 ^b^	0.14 ± 0.01 ^a^
L* (D65)	52.03 ± 0.53 ^a^	48.41 ± 0.11 ^b^	40.68 ± 0.80 ^c^
a* (D65)	13.78 ± 0.46 ^b^	14.52 ± 0.08 ^ab^	14.61 ± 0.31 ^a^
b* (D65)	35.64 ± 0.26 ^a^	33.68 ± 0.14 ^b^	30.03 ± 1.08 ^a^
ΔE	-	4.19	11.95
BI	47.97 ± 0.44^c^	51.60 ± 0.09 ^b^	59.32 ± 0.65 ^a^

Data are reported as SD mean and analyzed by one-way ANOVA followed by Tukey test for multiple comparisons. Different letters in row differ significantly with *p* < 0.05. Abbreviations: CTR, control trial; TPE5, taralli with 5% of PeP (w/w); TPE10 taralli with 10% of PeP (w/w).

**Table 3:** Cell viability of digested samples. Cells were treated for 24 h at the indicated dilution of 1:400, 1:200; 1:100; 1:150, 1:100, 1:50, and 1:25. Control (CTR−) cells were untreated (100% of vitality), and 90% of ethanol was used as a positive control (CTR+). Cells were then assayed for vitality by the calcein assay.

	CTR-	CTR+	CTR	TPE5	TPE10
1:400	1.00 ± 0.03 ^a^	0.42 ± 0.03 ^c^	1.01 ± 0.05 ^a^	0.92 ± 0.06 ^b^	0.96 ± 0.07 ^ab^
1:200	1.00 ± 0.02 ^a^	0.42 ± 0.03 ^c^	0.99 ± 0.04 ^a^	0.92 ± 0.04 ^b^	0.96 ± 0.07 ^ab^
1:150	1.00 ± 0.03 ^a^	0.42 ± 0.03 ^c^	1.01 ± 0.03 ^a^	0.88 ± 0.12 ^b^	0.99 ± 0.03 ^a^
1:100	1.00 ± 0.03 ^a^	0.42 ± 0.03 ^c^	0.97 ± 0.06 ^a^	0.87 ± 0.11 ^b^	0.98 ± 0.06 ^a^
1:50	1.00 ± 0.02 ^a^	0.42 ± 0.03 ^d^	0.98 ± 0.07 ^ab^	0.92 ± 0.06 ^bc^	0.90 ± 0.06 ^c^
1:25	1.00 ± 0.03 ^a^	0.42 ± 0.03 ^c^	0.82 ± 0.12 ^b^	0.83 ± 0.03 ^b^	0.86 ± 0.04 ^b^

Data are reported as SD mean and analyzed by one-way ANOVA followed by Tukey test for multiple comparisons. Different letters in row differ significantly with *p* < 0.05. Abbreviations: CTR, control trial; TPE5, taralli with 5% of PeP (w/w); TPE10 taralli with 10% of PeP (w/w).

## Data Availability

The data will be available upon request.

## Supplementary Materials

The following supporting information can be downloaded at: https://www.mdpi.com/article/10.3390/antiox14050550/s1. Figure S1: Original blot and gel related to Figure 5, showing the expression of proteins in HCT8 cells exposed with digested extract for 24 h for NFkB-pS536. Abbreviations: CTR, control trial; TPE5, taralli with 5% of Pep (w/w); TPE10 taralli with 10% of PeP (w/w); Figure S2: Original blot and gel related to Figure 5, showing the expression of proteins in HCT8 cells exposed with digested extract for 24 h for NFkB. Abbreviations: CTR, control trial; TPE5, taralli with 5% of Pep (w/w); TPE10 taralli with 10% of PeP (w/w); Figure S3: Original blot and gel related to Figure 6, showing the expression of proteins in HCT8 cells exposed with digested extract for 24 h for BID. Abbreviations: CTR, control trial; TPE5, taralli with 5% of Pep (w/w); TPE10 taralli with 10% of PeP (w/w).

## Abbreviations

The following abbreviations are used in this manuscript:

PeP	*Pleurotus eryngii* powder
ROS	Reactive oxygen species
TPA	textural profile analysis
TPC	total phenol content
GAE	Gallic acid equivalents
DPPH	2,2-diphenyl-1-picrylhydrazyl
ABTS	2,2′-azino-bis(3-ethylbenzothiazoline-6-sulfonic acid)
FRAP	Ferric reducing antioxidant power
TE	Trolox equivalents
HI	hydrolysis index
pGI	predicted glycaemic index
GP	gastric phase
DP	duodenal phase
BI	brown index
NFkB	nuclear factor kappa B
NFkB-pS536	phospho-nuclear factor kappa B at serine 536
BID	BH3 Interacting Domain Death Agonist
tBHP	tert-butyl hydroperoxide
SD	standard deviation
ISO	International Organization for Standardization
GI	glycemic index
TNF-α	Tumor necrosis factor-α
IL-1	Interleukin-10
IL-6	Interleukin-6
Bcl-2	B cell CLL/lymphoma-2
