# Supplementary figures and images for: Enhancing the Nutritional and Health-Related Properties of Taralli Through the Use of Pleurotus eryngii: Focus on Antioxidant and Anti-Inflammatory Properties
Source: Antioxidants (Basel). 2025 May 3;14(5):550. doi: 10.3390/antiox14050550 (PMC12108185; doi:10.3390/antiox14050550)

# NFkB-pS536

Figure S1

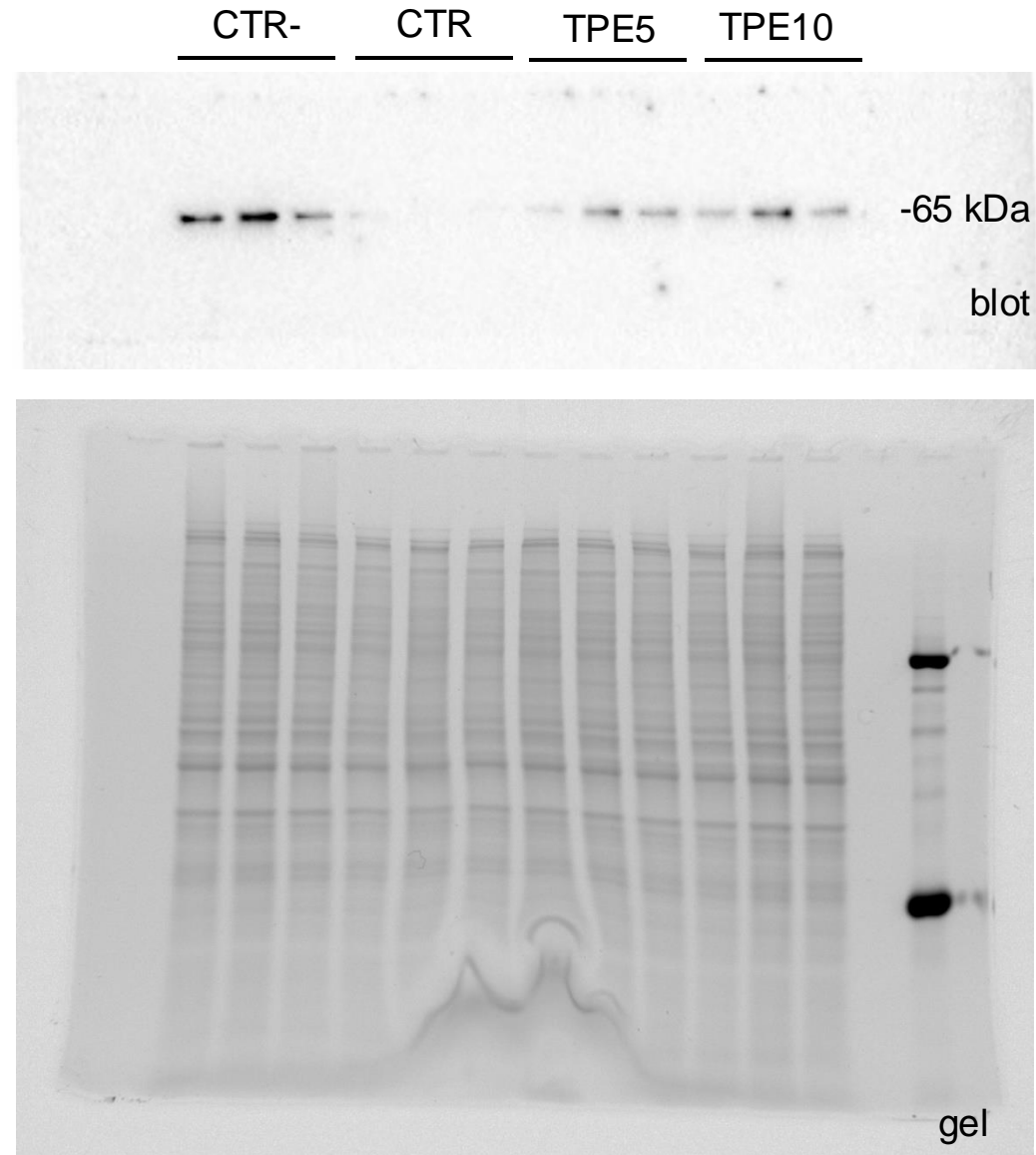

# NFkB

Figure S2

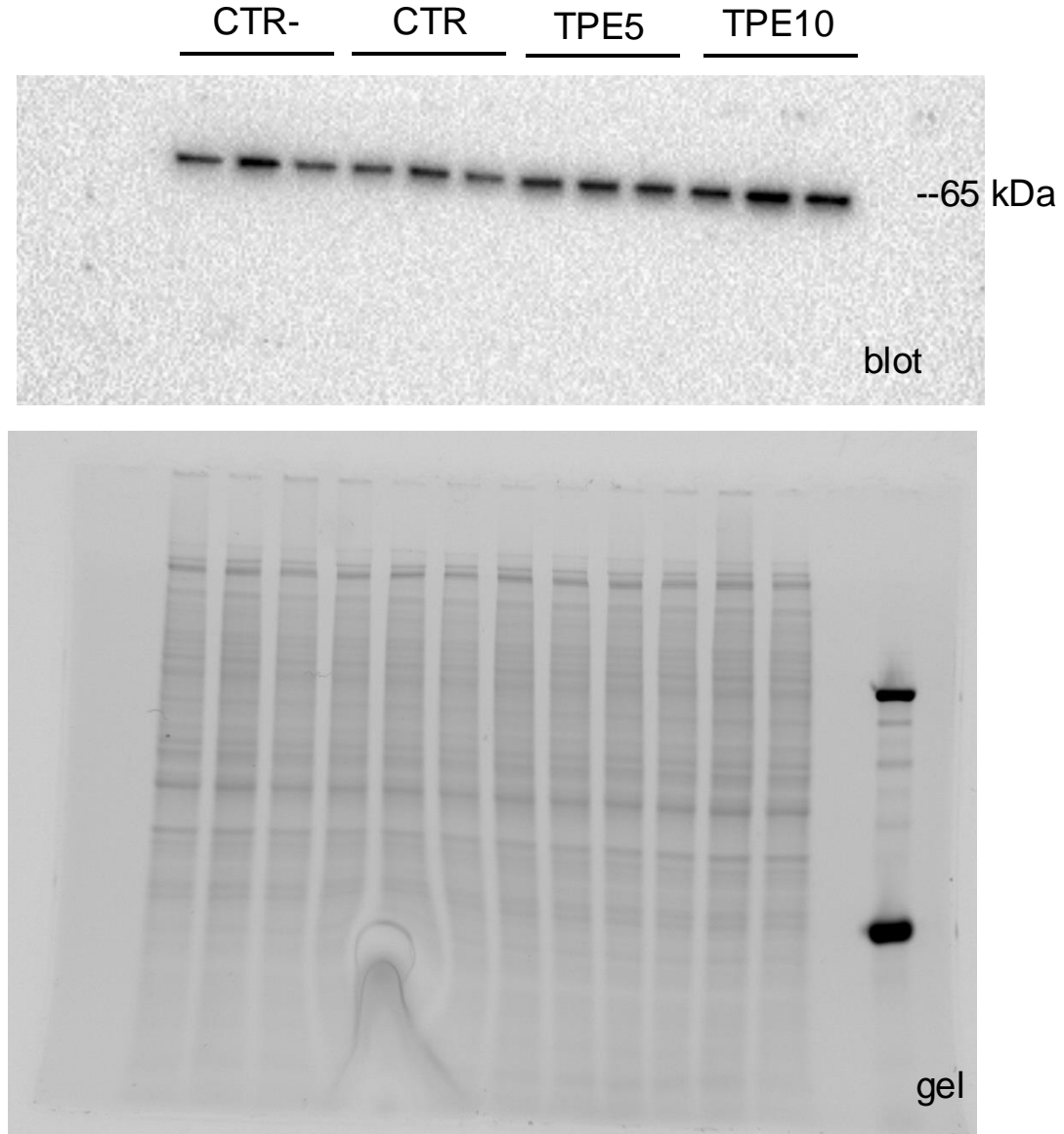

Figure S3

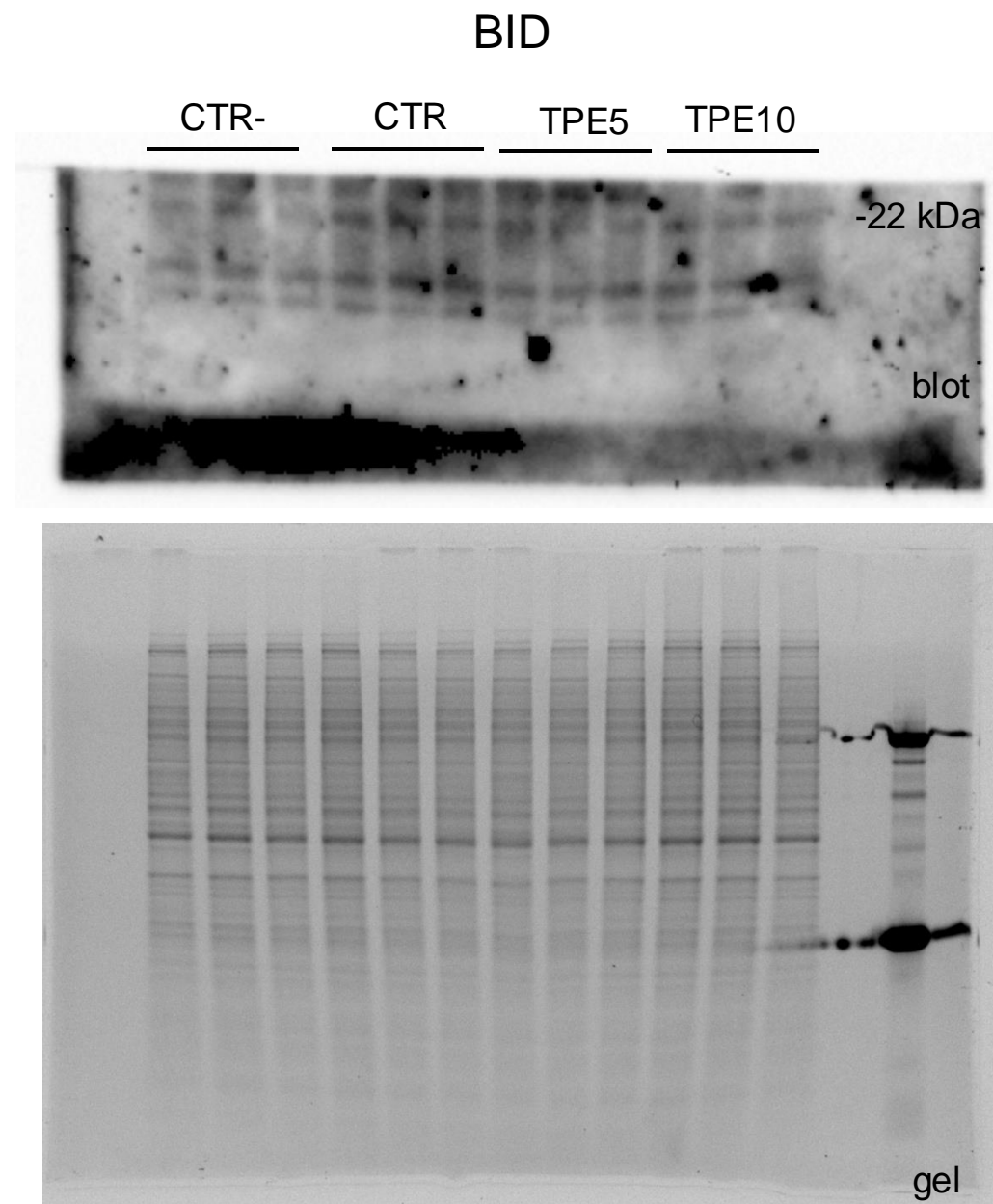

Supplement: Supplementary file 1 [file antioxidants-14-00550-s001.zip › antioxidants-3596658-supplementary.pdf]
